# Insula and Olfaction: A Literature Review and Case Report

**DOI:** 10.3390/brainsci11020198

**Published:** 2021-02-05

**Authors:** Frédérique Roy-Côté, Rayane Zahal, Johannes Frasnelli, Dang Khoa Nguyen, Olivier Boucher

**Affiliations:** 1 Département de Psychologie, Université de Montréal, Montréal, QC H3C 3J7, Canada; frederique.roy-cote@umontreal.ca (F.R.-C.); rayane.zahal@umontreal.ca (R.Z.); 2 Centre de Recherche du Centre Hospitalier de l’Université de Montréal, Montréal, QC H2X 0A9, Canada; d.nguyen@umontreal.ca; 3 Centre de Recherche de l’Hôpital du Sacré-Cœur-de-Montréal, Montréal, QC H4J 1C5, Canada; johannes.a.frasnelli@uqtr.ca; 4 Département d’anatomie, Université du Québec à Trois-Rivières, Trois-Rivières, QC G8Z 1V3, Canada; 5 Service de Neurologie, Centre Hospitalier de l’Université de Montréal, Montréal, QC H2X 3E4, Canada; 6 Département de Neurosciences, Université de Montréal, Montréal, QC H3C 3J7, Canada; 7 Service de Psychologie, Centre Hospitalier de l’Université de Montréal, Montréal, QC H2X 0C1, Canada

**Keywords:** insula, lesion, olfaction, Sniffin’ Sticks, cavernous angioma, review

## Abstract

(1) Background: It is well established that the insula is involved in olfaction, though its specific role in olfactory processing remains uncertain. In this paper, we first review the current literature on the insula and olfaction. Then, we describe the case of a 56-year-old man with a left insular cavernoma that caused olfactory disturbances. (2) Results: Structural neuroimaging studies suggest that insular gray matter volume is related to olfactory function, and functional neuroimaging shows that various types of stimuli lead to either lateralized or bilateral insular activations. Studies using electro-cortical stimulation reveal a specific region of the insular cortex, around the central insular sulcus, that could be related to unpleasant odor processing. Previous cases of insular lesions leading to olfactory disturbances suggest that left-sided insular lesions may more frequently lead to olfactory changes. In our patient with a left insular cavernoma, odors that were previously perceived as pleasant started smelling unpleasant and were hard to distinguish. Despite these subjective complaints, olfactory function assessed with the Sniffin’ Sticks test was normal. (3) Conclusions: Current tests may not be sensitive to all types of olfactory impairments associated with insular damage, and further studies should be conducted to develop olfactory tests assessing the hedonic appreciation of odors.

## 1. Introduction

The insula, often referred to as the fifth lobe of the brain, is a paralimbic structure located deep in the lateral sulcus. Its role in multisensory, affective, and cognitive processing is well documented [1,2,3,4,5,6,7]. Notably, cumulative evidence indicates that the insula is involved in olfaction. Tracing studies in primates and tractography in humans have shown connections between the primary olfactory cortex and the insula, which is proposed to be part of the secondary olfactory cortex [8,9,10]. A meta-analysis combining all published data on functional neuroimaging of olfaction identified the insula among the neural substrates of olfactory processing [11]. Furthermore, it is also known that olfaction is related to emotional processing, in which the insula plays an important role as well [12].

While it is established that the insula is involved in olfaction, its specific role in olfactory processing remains uncertain. This paper aims to further explore the insula’s role in olfaction by (1) reviewing the existing literature on the topic; and (2) presenting a new case of olfactory disturbance associated with an insular lesion.

## 2. Literature Review

The role of the insula in odor perception has been investigated using different approaches. Here, we present main findings.

### 2.1. Neuroimaging

#### 2.1.1. Structural Neuroimaging 

In individuals with a normal sense of smell, the cortical thickness of the insula is associated with olfactory quality discrimination in the right insula [13], but only in men. Another study on men showed that those with excellent results in smell tests had increased gray matter in the left anterior insula compared to participants with more regular results [14]. Similarly, master sommeliers showed higher gray matter volume in the right dorsal insula in comparison to a control group [15]. In turn, patients with different forms of olfactory dysfunction show significant insular gray matter loss: in the left anterior insula for patients with parosmia [16], the right insula for patients with chronic rhinosinusitis and severe olfactory dysfunction [17], and both insulae for patients with idiopathic olfactory loss [18]. In summary, these studies indicate that better olfactory function is associated with increased insular gray matter, whereas olfactory dysfunction is associated with decreased gray matter in this region.

#### 2.1.2. Functional Neuroimaging. 

The first study to observe cerebral regions involved in olfaction using functional neuroimaging found activations in the anterior insula bilaterally [19]. Several subsequent studies confirmed these results and observed insular activation when participants performed diverse olfactory tasks, such as odor discrimination [20,21], detecting a target odor within a mixture [22], odor naming [23], and odor imagery [24,25]. In contrast, other tasks such as odor detection [20] or odor recognition [21] were not associated with significant insular activation.

Stimulus valence appears to be key as the insula has been frequently associated with disgust processing [26,27]. For example, when being thirsty, water smelling fishy was perceived as less repulsive and led to significantly lower neural activity in the insular cortex than when participants were satiated [28]. On the other hand, the insula appears to be responsive to the quality of food odors: the sweeter the rating of a food odorant’s smell, the stronger the insular response [29]. In addition to valence, awareness appears to play an important role, as participants showed insular activations when they expected unpleasant stimuli, but not when they received it unexpectedly [30].

With regard to lateralized responses, the body of literature is less clear. Ambivalent odors frequently activated the right insula when participants perceived them as pleasant or unpleasant [31]. Some studies found pleasant odors to lead to greater left [32] or right [33] insular activity than unpleasant ones. Other studies found that disgusting odors particularly activated the right anterior insula [34]. Potentially linked to this, food odors activated the right insula significantly more than non-food odors [35].

In this context, it is important to note that the majority of odorants stimulate not only olfactory but also chemosensory receptors on the trigeminal nerve and the trigeminal system [36], leading to sensations such as burning (e.g., cinnamon), stinging (e.g., chili), or cooling (e.g., peppermint). CO2 (e.g., carbonated water) in higher concentrations is a potent stinging stimulus of the trigeminal system; more sensitive individuals exhibit higher activation in the insular cortex than less sensitive ones [37]. Again, valence appears to lead to lateralized responses: when combined with an orange odor, CO2 was generally perceived as relatively pleasant and activated the right insula; when combined with a rose odor, it was mostly perceived as unpleasant and led to activation of the left insula [38]. Mixed olfactory trigeminal stimuli led to stronger insular activations than pure olfactory stimuli, mainly on the right side for pleasant stimuli and bilaterally for neutral to unpleasant stimuli [39,40,41]. However, pure odors that are typically associated with trigeminal stimulation (e.g., trigeminal-free pepper essential oil) activate the insula to the same extent as the associated trigeminal stimulus (the odorless black pepper-derived pungent compound piperine), suggesting conditioning in the insular response [42].

In summary, these studies suggest that valence plays a crucial role for insular activation. Odors that are pleasant or unpleasant may also activate the insula differently on both sides.

### 2.2. Electro-Cortical Stimulation

Responses elicited by electrical stimulation of the insular cortex, in patients with epilepsy undergoing neurosurgery, provide additional crucial information on the role of the insula in sensory processing. Olfactory sensations following electrical stimulation of the insula have been described as “something funny (...), like medicine, a sickly smell” [43] and unpleasant [44,45], in line with the notion of phantosmia. It should be noted, however, that olfactory sensations represent only 1% of responses to electrical stimulation of the insular cortex and are concentrated around the central insular sulcus [44], while most responses are somatosensory and visceral sensations. Nevertheless, these findings are in line with phantom smells sometimes being part of epileptic auras in patients with insular epilepsy [46].

### 2.3. Previous Case Reports of Olfactory Dysfunction after Insular Lesion

Lesions restricted to the insula are very rare. In a study on 16 patients with insular lesions (following either a cerebrovascular accident, cavernoma, encephalitis, encephalomalacia, glioma, or tumor), 6/7 patients with left-sided insular lesions exhibited odor identification/intensity judgment, whereas 6/8 patients with right-sided lesions had no impairment. Still, the patients with apparently intact olfactory function performed worse than controls when carrying out tasks of odor similarity, odorant evaluations, and sweet odor discrimination [47]. In another report, involving patients with insular tumors, the authors found that 2/18 patients with right insular tumors reported olfactory sensations, while 0/22 patients with left insular tumors had such sensations [48].

Earlier, the case of a 59-year-old right-handed man who suffered several strokes within a fifteen-year span was described [49]. The first stroke was in the left insula, and led to no chemosensory impairment. Years later, the patient suffered from a second stroke, affecting the right insula. The patient reported that food had lost its taste and looked unappetizing. Four weeks later, the patient’s smell and taste had improved gradually, but not completely recovered. Tests showed that he could smell odors such as coffee grounds, vanilla, cloves, and perfume, but not fish or mint.

In another case study, a 70-year-old right-handed man with a stroke involving the posterior two thirds of the left insula and the supramarginal gyrus [50] reported heightened taste intensity; food, even if visually appealing, tasted intensely unpleasant and unfamiliar. He did not notice any changes in olfaction. During testing, the patient showed a heightened sensitivity to taste and odors, contralateral to the lesion, specifically to strong tastes (pleasant or unpleasant) and unpleasant odors.

More recently, a 55-year-old woman presented with phantosmia she described as “burned hair” after a stroke that affected the right posterior insula and the pre- and postcentral gyri [51]. The smell of burned hair disappeared after 24 h but the patient started suffering from mild hyposmia that was still present 6 months after the stroke. The patient also showed intermittent parosmia and a mild loss of taste.

Finally, a 61-year-old woman presented with focal seizures starting with olfacto-gustatory auras due to a low-grade glioma involving the left insula, anterior temporal lobe, and uncus [52]. After glioma resection, the patient reported complete loss of smell (anosmia) and taste (ageusia) that had not resolved even 3 years after the operation.

In summary, previous case studies suggest that left-sided insular lesions may be more frequently associated with olfactory changes, although these changes can be qualitatively different from one patient to another (i.e., heightened sensitivity to odors, partial or complete olfactory loss, parosmia, phantosmia). However, right-sided insular lesions may also cause olfactory disturbances such as phantosmia and hyposmia. Furthermore, a few cases suggest some specificity of olfactory symptoms for unpleasant odors.

## 3. Case Study

### 3.1. Methods

**Figure 1:**
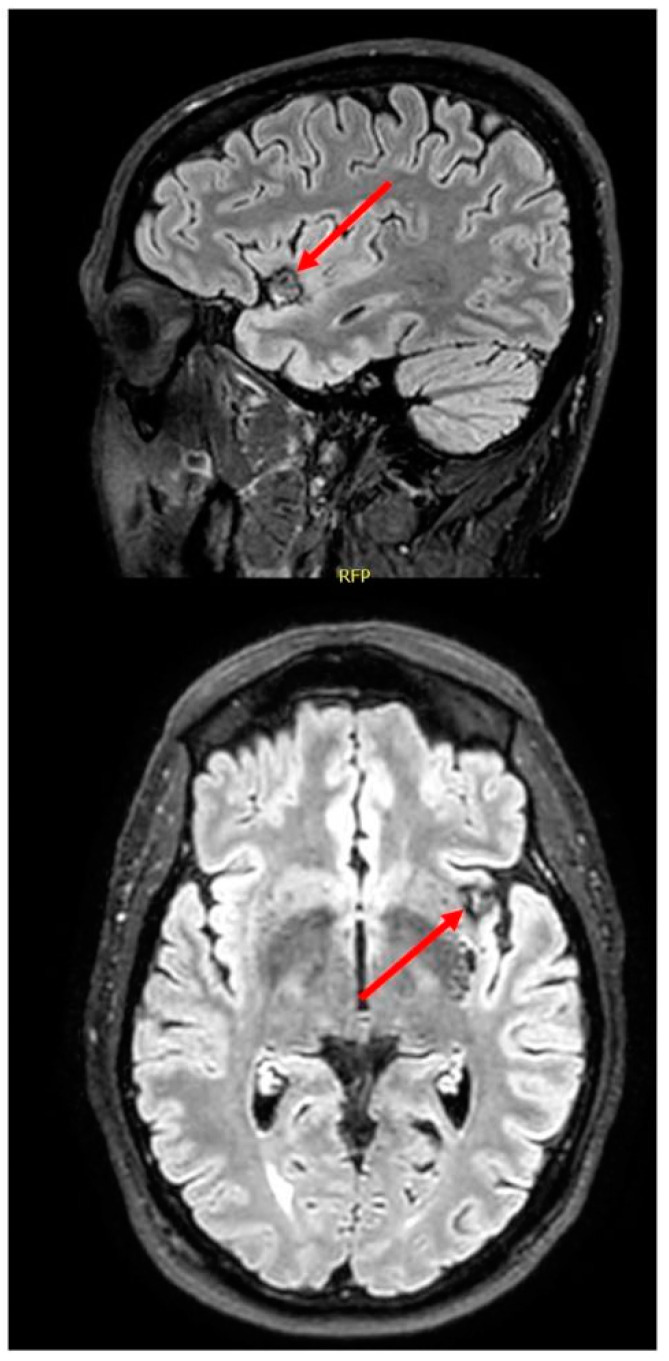
Brain magnetic resonance imaging (MRI) showing a left anterior insular cavernoma.

The patient is a 56-year-old right-handed engineer with no significant medical history except for hypercholesterolemia (treated with atorvastatin) and a possible mild concussion following a bicycle incident in 2015. In July of 2018, he noticed a change in his perception of odors, first with his perspiration and urine that he perceived as more intense than usual. Then, odors he found pleasant beforehand (e.g., spaghetti sauce, boiled vegetables) started smelling all the same and unpleasant. An unpleasant smell would not persist if he left the room but would last as long as he was exposed to it. He could not spontaneously describe this unpleasant smell but agreed when asked if it resembled decay or rot, among other options. He reported variations in the intensity of his disturbance with time (sometimes stronger, other times weaker) but claimed that it was always present. By contrast, the patient did not notice any change in appetite or taste and reported no weight loss. Examination by an ENT doctor was normal. Brain magnetic resonance imaging (MRI) ordered a year later by a neurologist disclosed a left insular cavernoma (Figure 1).

Olfactory and gustatory assessment was performed in February 2020 by a trained graduate student (R.Z.).

Olfactory testing: The Sniffin’ Sticks test battery [53] is a test of olfactory function assessing Threshold, Discrimination, and Identification. In the olfactory Threshold task, the blindfolded participant is asked to identify repeatedly a specific odor at different concentrations within non-odorant stimuli. Two sticks containing only a solvent are presented with one stick containing the odorant in different concentrations. The olfactory threshold is obtained using a standardized staircase procedure. The test yields a score between 1 (no detection threshold measurable) and 16 (lowest detection threshold). For odor Discrimination, 16 sets of three odorant stimuli are presented to the patient. Within these three odors, two are qualitatively identical and the other is different. In this task, the blindfolded participant is asked to identify the stimulus that is different from the other two. Lastly, for odor Identification, the participant is asked to identify 16 different olfactory stimuli (common odorants), among four choices (cued identification). In contrast to classical testing, which is carried out birhinally, Threshold and Identification tasks were tested separately for each nostril; for Identification, half the items were presented to the left nostril first, the other items were presented to the right nostril first, and then each item was presented back with the other nostril in a randomized order. Scores obtained in the individual tasks can be summed up to form a global score (TDI score for Threshold, Discrimination, and Identification) for which normative values are published [54,55].

We carried out two additional olfactory tasks. First, we asked the patient to spontaneously identify the odors before showing cues (spontaneous identification). Next, we asked the patient to rate each odor of the identification test for valence using a five-point scale from “−2” (very unpleasant) to “2” (very pleasant), “0” being “neutral”, adapted from the Self-Assessment Manikin [56].

Gustatory testing: The Taste Strips [57] is a psychophysical taste test used to measure gustatory function [58]. Using filter paper strips applied to the right or left side of the anterior third of the extended tongue, the participant has to identify different tastes with a multiple forced-choice of four descriptors (sweet, salty, sour, and bitter). The four taste qualities are presented in different concentrations, for a total of 32 trials. A “taste score” is obtained by summing up the number of correctly identified tastes. Moreover, for this test, normative scores for age groups and genders are published [58].

In addition, we carried out a basic neuropsychological assessment. Naming abilities in the visual modality were assessed using a shortened and French version of the Boston Naming Test with 30 items [59], and general cognitive screening was performed using the Montreal Cognitive Assessment (MoCA; Nasreddine, et al. [60]), administered by a licensed neuropsychologist (OB).

### 3.2. Results

Results obtained by our patient on olfactory and gustatory assessments are shown in Table 1. The patient’s olfactory threshold was normal for both nostrils when compared to his age–gender group. Odor discrimination, examined for both nostrils simultaneously, was within the average. Odor identification was somewhat weaker, being in the lower average. Without meeting the criteria for hyposmia, the patient had identification difficulties, particularly in the second half of the task; he seemed confused when stimuli were presented for the second time and tried to remember his first answer. Assessment of valence was not significantly different for the left (mean = 0.25, S.D. = 1.13) and right (mean = 0.50, S.D. = 0.97) nostrils (*t*_(15)_ = 1.29, *p* = 0.22). No odor was rated as strongly unpleasant; five were rated as somewhat unpleasant (−1) when presented at the left nostril (items #3, 4, 8, 9, and 14) and three at the right nostril (items #2, 3, and 9). Interestingly, one item (rose, #14) was rated as unpleasant (−1) when presented at the left nostril, but pleasant (+1) when presented at the right nostril, and the patient commented that the “rose” odorant used for the threshold task did not smell like rose and smelled bad.

For gustation, the patient’s results were within the average, on both sides. Cognitive screening performed on the same day as olfactory assessment revealed no evidence of neurocognitive disorder (MoCA = 28/30), and picture naming abilities were normal (abbreviated Boston Naming Test = 29/30). Furthermore, a comprehensive neuropsychological assessment performed eight months later revealed no significant cognitive deficits.

## 4. Discussion

In this article, we reviewed neuroimaging and electro-stimulation studies of the insula and olfaction, as well as previous case reports of olfactory dysfunction following insular lesions. We then described the case of a 56-year-old man with a left insular cavernoma and olfactory disturbances. Although several odors smelled the same and were unpleasant to him, olfactory testing did not show any significant olfactory deficit as the olfactory threshold, odor discrimination, and odor identification were all within the normal range (though somewhat weaker than the average). Assessment of odor valence did not reveal significant differences between both nostrils but allowed objectifying the patient’s complaints for a particular odorant (i.e., rose odor perceived as unpleasant).

Structural neuroimaging studies suggest that cortical measures of bilateral insulae are associated with olfactory sensitivity. While a thicker and denser cortex in healthy participants is associated with better olfactory performance [13,14,15], patients with different degrees and forms of olfactory dysfunction show loss of gray matter in the insula [16,17,18]. Functional neuroimaging studies show that the insula is activated to most olfactory stimuli and that hemispheric differences are associated with valence processing. While the right hemisphere may be specialized for pleasant stimuli, the left hemisphere may be more responsive to unpleasant stimuli across modalities [61,62]; this may also be true for olfactory stimuli. Some studies show analogous findings showing right insula activations to pleasant odorants or left insula activations to unpleasant ones [24,33,38,39,40]. However, some other studies show greater activation of the left insula to pleasant odors, or greater right insula activation to unpleasant ones [32,34], leaving this issue to be further explored. Electro-cortical stimulation studies of the insular cortex have reported olfactory responses which show some similarities to our case. Notably, these studies reported that stimulation of the insula more often causes unpleasant olfactory sensations [43,44,45]. Our patient described the unpleasant smell as resembling decay and rot, in line with parosmia. Similarly, patients from other studies described their olfactory sensations following insula stimulation as a sickly smell [43], or metallic/ether/chlorine [44]. A more specific association with unpleasant odors is congruent with the presumed role of the insula in disgust perception and processing [27,63,64,65].

The patient described in this paper experienced a change in his perception of odors: unpleasant odors smelled more intense, and odors that he found beforehand pleasant all smelled the same and unpleasant. Similar to our patient, an earlier case reported insular lesion causing heightened sensitivity to odors, specifically to unpleasant ones [50]. A case of unpleasant olfactory hallucinations of burned hair has also been described following insula lesions [51]. Additionally, previous cases of left insula lesions have led to olfactory disturbances, such as in our patient, including deranged smell, olfactory auras, and even anosmia [47,49,52]. Interestingly, while our patient had subjective complaints but no objective deficits in olfactory function, another case described in the literature noticed no subjective changes in olfaction while tests showed a heightened sensitivity to odors [50]. Therefore, anecdotal evidence seems to point at left insula lesions being responsible for olfactory disturbances, though manifestations and severity seem to vary widely across patients. Cases of olfactory disturbance following right insula lesions have also been described [47,48,49,51], but it remains unsure whether impairments caused by left and right insular lesions are qualitatively and quantitatively similar.

In our patient, smelling unpleasant odors more strongly may be explained by disturbed processing of unpleasant odors due to impaired modulation of these stimuli, or conversely by impaired ability to process pleasantness in odors. Interestingly, insular lesions have also previously been associated with increased sensitivity to loud sounds [66] and with increased sensitivity to noxious stimuli [67], in line with the modulation hypothesis.

Despite significant olfactory complaints in our patient, formal olfactory assessment revealed no deficit, which may indicate that the test we used, i.e., the Sniffin’ Sticks test, may not be sensitive to all types of olfactory impairments associated with insular damage. Normative data on subjective ratings of valence of odorants would have allowed us to test whether our patient’s qualitative and hedonic appreciation of odorants is disturbed, beyond odorant detection and identification. Therefore, development of such normative data would be needed in order to better assess olfactory changes associated with differing etiologies. Nevertheless, a sudden change in the hedonic perception of odorants could hint to an insular lesion and thus allow for earlier clinical management by a neurologist.

## Figures and Tables

**Table 1:** Results obtained by our patient on olfactory and gustatory tests.

	Raw Score	Percentile	Interpretation
Olfactory Threshold			
Left nostril	5.25	25–50	Average
Right nostril	5.75	10–25	Low average
Odor Discrimination			
Both nostrils	11	25	Low average
Odor Identification			
Left nostril	11	25	Low average
Right nostril	10	10–25	Low average
TDI composite score			
Left nostril	27.25	-	-
Right nostril	26.75	-	-
Taste Strips			
Left side	8	25	Low average
Right side	10	25–50	Average

Note. Interpretation according to normative data for the olfactory Threshold, Discrimination, and Identification subtests from the Sniffin’ Sticks test battery [55] and for the Taste Strips [58].
